# Supplementary material for: Association of Plant-Based Diet Indices and Abdominal Obesity with Mental Disorders among Older Chinese Adults
Source: Nutrients. 2023 Jun 12;15(12):2721. doi: 10.3390/nu15122721 (PMC10303527; doi:10.3390/nu15122721)
Supplement: Supplementary file 1 [file nutrients-15-02721-s001.zip › nutrients-2368982-supplementary.pdf]

Supplementary material

Contents

Table S1. Scoring of plant-based diet indices. 1

Table S2. Multiplicative interaction between three plant diet indices and depression. 3

Table S3. Multiplicative interaction between the three plant diet indices and anxiety. 3

Table S4. Association of three plant-based diet indices with depression and anxiety, stratified by sex. 4

Table S5. Association of abdominal obesity with depression and anxiety, stratified by sex. ... 5

Table S6. Association of three plant-based diet indices with depression and anxiety, stratified by age group. 6

Table S7. Association of abdominal obesity with depression and anxiety, stratified by age group. 7

Table S8. Association of three plant-based diet indices with depression and anxiety, stratified by residence. 8

Table S9. Association of abdominal obesity with depression and anxiety, stratified by residence. 9

Table S10. Association of three plant-based diet indices with depression and anxiety, stratified by economic situation. 10

Table S11. Association of abdominal obesity with depression and anxiety, stratified by economic situation. 11

Table S12. Association of three plant-based diet indices with depression and anxiety, stratified by comorbidity. 12

Table S13. Association of abdominal obesity with depression and anxiety, stratified by comorbidity. 13

Table S1. Scoring of plant-based diet indices.

Types	Food	Frequency	PDI	hPDI	uPDI
Healthful plant-based food	Whole grain	Yes	5	5	1
		No	1	1	5
	Vegetable oil	Yes	5	5	1
		No	1	1	5
	Fresh fruit	Almost everyday	5	5	1
		Except winter	4	4	2
		Occasionally	2	2	4
		Rarely or never	1	1	5
	Vegetable	Almost everyday	5	5	1
		Except winter	4	4	2
		Occasionally	2	2	4
		Rarely or never	1	1	5
	Legume	Almost everyday	5	5	1
		≥1 time/week	4	4	2
		≥1 time/month	3	3	3
		Occasionally	2	2	4
		Rarely or never	1	1	5
	Garlic	Almost everyday	5	5	1
		≥1 time/week	4	4	2
		≥1 time/month	3	3	3
		Occasionally	2	2	4
		Rarely or never	1	1	5
	Nut products	Almost everyday	5	5	1
		≥1 time/week	4	4	2
		≥1 time/month	3	3	3
		Occasionally	2	2	4
		Rarely or never	1	1	5
	Tea	Almost everyday	5	5	1
		≥1 time/week	4	4	2
		≥1 time/month	3	3	3
		Occasionally	2	2	4
		Rarely or never	1	1	5
Unhealthful plant-based food	Refined grain	Yes	5	1	5
		No	1	5	1
	Salt-preserved vegetable	Almost everyday	5	1	5
		≥1 time/week	4	2	4
		≥1 time/month	3	3	3
		Occasionally	2	4	2
		Rarely or never	1	5	1

	Sugar	Almost everyday	5	1	5
		≥1 time/week	4	2	4
		≥1 time/month	3	3	3
		Occasionally	2	4	2
		Rarely or never	1	5	1
Animal-based food	Animal fat	Yes	1		
		No	5		
	Meat	Almost everyday	1		
		≥1 time/week	2		
		≥1 time/month	3		
		Occasionally	4		
		Rarely or never	5		
	Fish	Almost everyday	1		
		≥1 time/week	2		
		≥1 time/month	3		
		Occasionally	4		
		Rarely or never	5		
	Egg	Almost everyday	1		
		≥1 time/week	2		
		≥1 time/month	3		
		Occasionally	4		
		Rarely or never	5		
	Milk products	Almost everyday	1		
		≥1 time/week	2		
		≥1 time/month	3		
		Occasionally	4		
		Rarely or never	5		

Table S2. Multiplicative interaction between three plant diet indices and depression.

Abdominal obesity	PDI	OR (95% CI)	*p*-Value	hPDI	OR (95% CI)	*p*-Value	uPDI	OR (95% CI)	*p*-Value
0	Q1	1.00		Q1	1.00(Ref.)		Q4	1.00(Ref.)	
1	Q1	0.78(0.67-0.91)	< 0.01	Q1	0.80(0.68-0.95)	< 0.01	Q4	0.82(0.69-0.98)	< 0.05
0	Q2	0.78(0.65-0.94)	< 0.01	Q2	0.75(0.63-0.89)	< 0.001	Q3	0.75(0.62-0.90)	< 0.01
1	Q2	0.71(0.60-0.84)	< 0.001	Q2	0.70(0.59-0.82)	< 0.001	Q3	0.64(0.54-0.77)	< 0.001
0	Q3	0.73(0.61-0.87)	< 0.001	Q3	0.69(0.57-0.85)	< 0.001	Q2	0.68(0.56-0.83)	< 0.001
1	Q3	0.60(0.51-0.71)	< 0.001	Q3	0.58(0.49-0.70)	< 0.001	Q2	0.52(0.43-0.63)	< 0.001
0	Q4	0.52(0.42-0.65)	< 0.001	Q4	0.59(0.48-0.73)	< 0.001	Q1	0.57(0.46-0.71)	< 0.001
1	Q4	0.53(0.44-0.64)	< 0.001	Q4	0.52(0.43-0.62)	< 0.001	Q1	0.57(0.47-0.68)	< 0.001

Participants with abdominal obesity were assigned a value of 1, and those without abdominal obesity were assigned a value of 0.

Table S3. Multiplicative interaction between the three plant diet indices and anxiety.

Abdominal obesity	PDI	OR (95% CI)	*p*-Value	hPDI	OR (95% CI)	*p*-Value	uPDI	OR (95% CI)	*p*-Value
0	Q1	1.00		Q1	1.00(Ref.)		Q4	1.00(Ref.)	
1	Q1	0.83(0.67-1.02)	0.079	Q1	0.70(0.57-0.87)	< 0.01	Q4	0.84(0.68-1.06)	0.137
0	Q2	1.02(0.80-1.30)	0.862	Q2	0.72(0.58-0.90)	< 0.01	Q3	0.90(0.71-1.13)	0.353
1	Q2	0.67(0.53-0.85)	< 0.001	Q2	0.59(0.47-0.73)	< 0.001	Q3	0.66(0.52-0.83)	< 0.001
0	Q3	0.85(0.67-1.07)	0.163	Q3	0.70(0.54-0.91)	< 0.01	Q2	0.69(0.53-0.90)	< 0.01
1	Q3	0.68(0.54-0.85)	< 0.001	Q3	0.55(0.43-0.70)	< 0.001	Q2	0.53(0.41-0.69)	< 0.001
0	Q4	0.77(0.58-1.02)	0.067	Q4	0.54(0.40-0.71)	< 0.001	Q1	0.68(0.52-0.90)	< 0.01
1	Q4	0.69(0.54-0.88)	< 0.01	Q4	0.54(0.42-0.68)	< 0.001	Q1	0.55(0.43-0.71)	< 0.001

Participants with abdominal obesity were assigned a value of 1, and those without abdominal obesity were assigned a value of 0.

Table S4. Association of three plant-based diet indices with depression and anxiety, stratified by sex.

	Q1		Q2			Q3			Q4		
	Case/total	OR (95% CI)	Case/total	OR (95% CI)	*p*-Value	Case/total	OR (95% CI)	*p*-Value	Case/total	OR (95% CI)	*p*-Value
**Depression**											
Male											
PDI	418/1521	1.00	276/1130	0.95(0.78-1.14)	0.570	327/1492	0.86(0.72-1.03)	0.093	192/1283	0.64(0.52-0.78)	< 0.001
hPDI	366/1301	1.00	381/1578	0.92(0.77-1.10)	0.382	253/1233	0.81(0.66-0.98)	< 0.05	213/1314	0.65(0.53-0.79)	< 0.001
uPDI	298/1883	1.00	248/1186	1.15(0.94-1.41)	0.175	324/1409	1.13(0.93-1.39)	0.223	343/948	1.77(1.43-2.20)	< 0.001
Female											
PDI	773/2016	1.00	437/1390	0.79(0.68-0.93)	< 0.01	450/1648	0.69(0.59-0.80)	< 0.001	267/1143	0.61(0.51-0.73)	< 0.001
hPDI	671/1765	1.00	591/1901	0.75(0.65-0.87)	< 0.001	346/1275	0.66(0.55-0.78)	< 0.001	319/1256	0.61(0.52-0.73)	< 0.001
uPDI	381/1579	1.00	330/1283	0.90(0.75-1.09)	0.280	577/1746	1.21(1.01-1.44)	< 0.05	639/1589	1.41(1.18-1.69)	< 0.001
**Anxiety**											
Male											
PDI	142/1521	1.00	106/1130	1.09(0.83-1.44)	0.523	143/1492	1.14(0.88-1.47)	0.319	91/1283	0.92(0.69-1.23)	0.579
hPDI	136/1301	1.00	133/1578	0.82(0.63-1.07)	0.143	111/1233	0.92(0.70-1.21)	0.544	102/1314	0.80(0.60-1.06)	0.122
uPDI	136/1883	1.00	86/1186	0.88(0.65-1.19)	0.410	137/1409	1.11(0.84-1.47)	0.481	123/948	1.24(0.91-1.68)	0.170
Female											
PDI	349/2016	1.00	192/1390	0.81(0.67-0.99)	< 0.05	196/1648	0.70(0.58-0.85)	< 0.001	142/1143	0.78(0.63-0.98)	< 0.05
hPDI	310/1765	1.00	272/1901	0.77(0.64-0.93)	< 0.01	153/1275	0.66(0.53-0.82)	< 0.001	1441256	0.60(0.48-0.76)	< 0.001
uPDI	158/1579	1.00	149/1283	1.08(0.84-1.39)	0.537	261/1746	1.35(1.07-1.71)	0.010	311/1589	1.67(1.32-2.12)	< 0.001

Estimates are odds ratios (95% confidence intervals) from multivariable logistic regression models adjusted for age, residence, marital status, cohabitation status, education, occupation, economic situation, sleep duration, smoking status, alcohol consumption, physical exercise, leisure activity participation status, BMI, chronic disease and comorbidity.

Table S5. Association of abdominal obesity with depression and anxiety, stratified by sex.

	Non-abdominal obesity		Abdominal obesity		
	Case/total	OR (95% CI)	Case/total	OR (95% CI)	*p*-Value
**Male**					
Depression	605/2321	1.00	608/3105	0.75(0.65-0.86)	< 0.001
Anxiety	238/2321	1.00	244/3105	0.76(0.62-0.93)	< 0.01
**Female**					
Depression	707/2042	1.00	1220/4155	0.83(0.73-0.94)	< 0.01
Anxiety	336/2042	1.00	543/4155	0.74(0.64-0.87)	< 0.001

Estimates are odds ratios (95% confidence intervals) from multivariable logistic regression models adjusted for age, residence, marital status, cohabitation status, education, occupation, economic situation, sleep duration, smoking status, alcohol consumption, physical exercise, leisure activity participation status, chronic disease and comorbidity.

Table S6. Association of three plant-based diet indices with depression and anxiety, stratified by age group.

	Q1		Q2			Q3			Q4		
	Case/total	OR (95% CI)	Case/total	OR (95% CI)	*p*-Value	Case/total	OR (95% CI)	*p*-Value	Case/total	OR (95% CI)	*p*-Value
**Depression**											
≤80 years old											
PDI	364/1234	1.00	256/1026	0.87(0.71-1.06)	0.157	324/1530	0.72(0.60-0.87)	0.001	240/1326	0.69(0.57-0.85)	< 0.001
hPDI	301/988	1.00	389/1519	0.85(0.71-1.03)	0.102	225/1183	0.62(0.52-0.77)	< 0.001	269/1426	0.65(0.53-0.80)	< 0.001
uPDI	311/1807	1.00	225/1100	1.08(0.88-1.33)	0.473	317/1284	1.22(1.00-1.50)	0.054	331/925	1.71(1.38-2.12)	< 0.001
> 80 years old											
PDI	827/2303	1.00	457/1494	0.85(0.73-0.99)	< 0.05	453/1610	0.79(0.68-0.91)	0.001	219/1100	0.55(0.46-0.66)	< 0.001
hPDI	736/2078	1.00	583/1960	0.79(0.69-0.91)	0.001	374/1325	0.78(0.67-0.92)	< 0.01	263/1144	0.60(0.50-0.71)	< 0.001
uPDI	368/1655	1.00	353/1369	0.97(0.81-1.16)	0.731	584/1871	1.16(0.97-1.38)	0.099	651/1612	1.46(1.22-1.75)	< 0.001
**Anxiety**											
≤80 years old											
PDI	186/1234	1.00	138/1026	0.93(0.73-1.19)	0.566	171/1530	0.79(0.62-0.99)	< 0.05	140/1326	0.83(0.65-1.07)	0.144
hPDI	146/988	1.00	202/1519	0.93(0.73-1.18)	0.533	138/1183	0.86(0.66-1.11)	0.241	149/1426	0.75(0.58-0.97)	< 0.05
uPDI	169/1807	1.00	106/1100	0.93(0.71-1.21)	0.571	175/1284	1.27(0.99-1.64)	0.064	185/925	1.67(1.28-2.18)	< 0.001
> 80 years old											
PDI	305/2303	1.00	160/1494	0.86(0.70-1.06)	0.160	168/1610	0.86(0.70-1.06)	0.160	93/1100	0.76(0.59-0.98)	< 0.05
hPDI	300/2078	1.00	203/1960	0.70(0.58-0.85)	< 0.001	126/1335	0.68(0.54-0.85)	0.001	97/1144	0.60(0.47-0.78)	< 0.001
uPDI	125/1655	1.00	129/1369	1.04(0.79-1.36)	0.791	223/1871	1.23(0.96-1.59)	0.106	249/1612	1.40(1.08-1.81)	< 0.05

Estimates are odds ratios (95% confidence intervals) from multivariable logistic regression models adjusted for sex, residence, marital status, cohabitation status, education, occupation, economic situation, sleep duration, smoking status, alcohol consumption, physical exercise, leisure activity participation status, BMI, chronic disease and comorbidity.

Table S7. Association of abdominal obesity with depression and anxiety, stratified by age group.

	Non-abdominal obesity		Abdominal obesity		
	Case/total	OR (95% CI)	Case/total	OR (95% CI)	*p*-Value
**≤80 years old**					
Depression	381/1566	1.00	803/3550	0.89(0.77-1.04)	0.145
Anxiety	219/1566	1.00	416/3550	0.73(0.61-0.88)	0.001
**＞80 years old**					
Depression	931/2797	1.00	1025/3710	0.74(0.66-0.83)	< 0.001
Anxiety	355/2797	1.00	371/3710	0.75(0.64-0.88)	0.001

Estimates are odds ratios (95% confidence intervals) from multivariable logistic regression models adjusted for sex, residence, marital status, cohabitation status, education, occupation, economic situation, sleep duration, smoking status, alcohol consumption, physical exercise, leisure activity participation status, chronic disease and comorbidity.

Table S8. Association of three plant-based diet indices with depression and anxiety, stratified by residence.

	Q1		Q2			Q3			Q4		
	Case/total	OR (95% CI)	Case/total	OR (95% CI)	*p*-Value	Case/total	OR (95% CI)	*p*-Value	Case/total	OR (95% CI)	*p*-Value
**Depression**											
Urban											
PDI	177/660	1.00	174/627	1.09(0.83-1.43)	0.545	155/762	0.79(0.60-1.04)	0.095	109/694	0.68(0.50-0.91)	< 0.01
hPDI	174/555	1.00	212/831	0.78(0.60-1.02)	0.065	122/660	0.59(0.44-0.79)	< 0.001	107/697	0.53(0.39-0.72)	< 0.001
uPDI	324/1613	1.00	133/550	1.02(0.79-1.32)	0.853	97/393	1.11(0.83-1.49)	0.486	61/187	1.56(1.06-2.28)	< 0.05
Town											
PDI	1014/2877	1.00	539/1893	0.80(0.70-0.91)	0.001	622/2378	0.75(0.66-0.85)	< 0.001	350/1732	0.61(0.53-0.71)	< 0.001
hPDI	863/2511	1.00	760/2648	0.81(0.72-0.92)	0.001	477/1848	0.75(0.65-0.87)	< 0.001	425/1873	0.66(0.57-0.76)	< 0.001
uPDI	355/1849	1.00	445/1919	1.03(0.87-1.21)	0.770	804/2762	1.22(1.05-1.43)	0.010	921/2350	1.59(1.36-1.86)	< 0.001
**Anxiety**											
Urban											
PDI	76/660	1.00	63/627	0.88(0.61-1.28)	0.511	56/762	0.66(0.45-0.96)	< 0.05	50/694	0.73(0.49-1.09)	0.128
hPDI	74/555	1.00	71/831	0.64(0.45-0.92)	< 0.05	51/660	0.63(0.43-0.94)	< 0.05	49/697	0.58(0.39-0.88)	< 0.01
uPDI	116/1613	1.00	63/550	1.52(1.08-2.14)	< 0.05	40/393	1.39(0.93-2.09)	0.108	26/187	1.77(1.07-2.92)	< 0.05
Town											
PDI	415/2877	1.00	235/1893	0.89(0.75-1.07)	0.204	283/2378	0.87(0.74-1.30)	0.107	183/1732	0.83(0.68-1.01)	0.059
hPDI	372/2511	1.00	334/2648	0.82(0.69-0.96)	< 0.05	213/1848	0.77(0.64-0.93)	< 0.01	197/1873	0.68(0.56-0.83)	< 0.001
uPDI	178/1849	1.00	172/1919	0.83(0.66-1.05)	0.117	358/2762	1.16(0.95-1.42)	0.159	408/2350	1.37(1.12-1.68)	< 0.01

Estimates are odds ratios (95% confidence intervals) from multivariable logistic regression models adjusted for age, sex, marital status, cohabitation status, education, occupation, economic situation, sleep duration, smoking status, alcohol consumption, physical exercise, leisure activity participation status, BMI, chronic disease and comorbidity.

Table S9. Association of abdominal obesity with depression and anxiety, stratified by residence.

	Non-abdominal obesity		Abdominal obesity		
	Case/total	OR (95% CI)	Case/total	OR (95% CI)	*p*-Value
**Urban**					
Depression	216/829	1.00	399/1914	0.76(0.61-0.94)	< 0.05
Anxiety	91/829	1.00	154/1914	0.69(0.52-0.92)	< 0.05
**Town**					
Depression	1096/3534	1.00	1429/5346	0.81(0.73-0.90)	< 0.001
Anxiety	483/3534	1.00	633/5334	0.76(0.66-0.87)	< 0.001

Estimates are odds ratios (95% confidence intervals) from multivariable logistic regression models adjusted for age, sex, marital status, cohabitation status, education, occupation, economic situation, sleep duration, smoking status, alcohol consumption, physical exercise, leisure activity participation status, chronic disease and comorbidity.

Table S10. Association of three plant-based diet indices with depression and anxiety, stratified by economic situation.

	Q1		Q2			Q3			Q4		
	Case/total	OR (95% CI)	Case/total	OR (95% CI)	*p*-Value	Case/total	OR (95% CI)	*p*-Value	Case/total	OR (95% CI)	*p*-Value
**Depression**											
Not wealthy											
PDI	1075/2960	1.00	622/1997	0.84(0.74-0.95)	< 0.01	666/2423	0.73(0.65-0.83)	< 0.001	404/1819	0.63(0.55-0.73)	< 0.001
hPDI	930/2569	1.00	839/2736	0.80(0.71-0.90)	< 0.001	529/1911	0.73(0.64-0.84)	< 0.001	469/1983	0.63(0.54-0.72)	< 0.001
uPDI	540/2409	1.00	507/1909	1.06(0.91-1.23)	0.465	802/2616	1.21(1.05-1.40)	0.010	918/2265	1.60(1.38-1.86)	< 0.001
Wealthy											
PDI	116/577	1.00	91/523	0.97(0.70-1.33)	0.827	111/717	0.90(0.66-1.23)	0.516	55/607	0.51(0.36-0.74)	< 0.001
hPDI	107/497	1.00	133/743	0.92(0.68-1.25)	0.581	70/597	0.59(0.42-0.84)	< 0.01	63/587	0.56(0.39-0.81)	< 0.01
uPDI	139/1053	1.00	71/560	0.85(0.61-1.19)	0.351	99/539	1.15(0.83-1.60)	0.401	64/272	1.22(0.82-1.80)	0.332
**Anxiety**											
Not wealthy											
PDI	445/2960	1.00	263/1997	0.89(0.75-1.05)	0.173	292/2423	0.82(0.69-0.96)	< 0.05	202/1819	0.81(0.67-0.98)	< 0.05
hPDI	396/2569	1.00	358/2736	0.80(0.68-0.94)	< 0.01	235/1911	0.77(0.65-0.93)	< 0.01	213/1983	0.67(0.55-0.80)	< 0.001
uPDI	228/2409	1.00	203/1909	1.03(0.84-1.27)	0.764	359/2616	1.31(1.08-1.60)	< 0.01	412/2265	1.60(1.31-1.96)	< 0.001
Wealthy											
PDI	46/577	1.00	35/523	0.91(0.57-1.45)	0.685	47/717	0.92(0.59-1.43)	0.714	31/607	0.75(0.45-1.22)	0.242
hPDI	50/497	1.00	47/743	0.68(0.44-1.05)	0.079	29/597	0.54(0.33-0.88)	< 0.05	33/587	0.62(0.38-1.01)	0.053
uPDI	66/1053	1.00	32/560	0.83(0.52-1.31)	0.424	39/539	0.98(0.62-1.55)	0.920	22/272	0.86(0.48-1.52)	0.600

Estimates are odds ratios (95% confidence intervals) from multivariable logistic regression models adjusted for age, sex, residence, marital status, cohabitation status, education, occupation, sleep duration, smoking status, alcohol consumption, physical exercise, leisure activity participation status, BMI, chronic disease and comorbidity.

Table S11. Association of abdominal obesity with depression and anxiety, stratified by economic situation.

	Non-abdominal obesity		Abdominal obesity		
	Case/total	OR (95% CI)	Case/total	OR (95% CI)	*p*-Value
**Not wealthy**					
Depression	1157/3564	1.00	1610/5635	0.82(0.74-0.91)	< 0.001
Anxiety	502/3564	1.00	700/5635	0.79(0.69-0.90)	< 0.001
**Wealthy**					
Depression	155/799	1.00	218/1625	0.63(0.49-0.80)	< 0.001
Anxiety	72/799	1.00	87/1625	0.52(0.37-0.73)	< 0.001

Estimates are odds ratios (95% confidence intervals) from multivariable logistic regression models adjusted for age, sex, residence, marital status, cohabitation status, education, occupation, sleep duration, smoking status, alcohol consumption, physical exercise, leisure activity participation status, chronic disease and comorbidity.

Table S12. Association of three plant-based diet indices with depression and anxiety, stratified by comorbidity.

	Q1		Q2			Q3			Q4		
	Case/total	OR (95% CI)	Case/total	OR (95% CI)	*p*-Value	Case/total	OR (95% CI)	*p*-Value	Case/total	OR (95% CI)	*p*-Value
**Depression**											
Comorbidity											
PDI	281/759	1.00	226/674	0.96(0.76-1.22)	0.758	256/846	0.92(0.73-1.15)	0.453	175/668	0.82(0.64-1.06)	0.126
hPDI	239/617	1.00	305/899	0.92(0.73-1.16)	0.499	197/690	0.78(0.60-1.00)	< 0.05	197/741	0.76(0.59-0.98)	< 0.05
uPDI	317/1169	1.00	195/645	0.93(0.73-1.17)	0.521	193/607	0.91(0.71-1.17)	0.459	233/526	1.31(1.01-1.70)	< 0.05
Non-comorbidity											
PDI	910/2778	1.00	487/1846	0.83(0.72-0.95)	< 0.01	521/2294	0.70(0.61-0.80)	< 0.001	284/1758	0.55(0.47-0.65)	< 0.001
hPDI	798/2449	1.00	667/2580	0.79(0.69-0.90)	< 0.001	402/1818	0.70(0.61-0.82)	< 0.001	335/1829	0.59(0.50-0.68)	< 0.001
uPDI	362/2293	1.00	383/1824	1.08(0.91-1.28)	0.375	708/2548	1.33(1.13-1.55)	< 0.001	749/2011	1.70(1.44-2.01)	< 0.001
**Anxiety**											
Comorbidity											
PDI	120/759	1.00	101/674	1.05(0.78-1.42)	0.746	114/846	0.98(0.73-1.32)	0.894	78/668	0.88(0.64-1.22)	0.440
hPDI	99/617	1.00	127/899	0.89(0.66-1.20)	0.427	90/690	0.87(0.63-1.20)	0.384	97/741	0.87(0.63-1.20)	0.395
uPDI	114/1169	1.00	84/645	1.15(0.84-1.59)	0.378	96/607	1.30(0.94-1.80)	0.109	119/526	1.66(1.19-2.31)	< 0.01
Non-comorbidity											
PDI	371/2778	1.00	197/1846	0.84(0.70-1.01)	0.069	225/2294	0.78(0.65-0.94)	< 0.01	155/1758	0.79(0.64-0.98)	< 0.05
hPDI	347/2449	1.00	278/2580	0.75(0.63-0.90)	0.001	174/1818	0.70(0.57-0.86)	0.001	149/1829	0.59(0.48-0.73)	< 0.001
uPDI	180/2293	1.00	151/1824	0.91(0.72-1.16)	0.458	302/2548	1.20(0.97-1.49)	0.092	315/2011	1.42(1.14-1.77)	< 0.01

Estimates are odds ratios (95% confidence intervals) from multivariable logistic regression models adjusted for age, sex, residence, marital status, cohabitation status, education, occupation, economic situation, sleep duration, smoking status, alcohol consumption, physical exercise, leisure activity participation status, BMI and chronic disease.

Table S13. Association of abdominal obesity with depression and anxiety, stratified by comorbidity.

	Non-abdominal obesity		Abdominal obesity		
	Case/total	OR (95% CI)	Case/total	OR (95% CI)	*p*-Value
**Comorbidity**					
Depression	303/762	1.00	635/2185	0.67(0.56-0.81)	< 0.001
Anxiety	134/762	1.00	279/2185	0.67(0.53-0.85)	0.001
**Non-comorbidity**					
Depression	1009/3601	1.00	1193/5075	0.84(0.76-0.93)	0.001
Anxiety	440/3601	1.00	508/5075	0.78(0.67-0.90)	< 0.001

Estimates are odds ratios (95% confidence intervals) from multivariable logistic regression models adjusted for age, sex, residence, marital status, cohabitation status, education, occupation, economic situation, sleep duration, smoking status, alcohol consumption, physical exercise, leisure activity participation status and chronic disease.
